# An Analysis of the Impact of the Emissions Trading System on the Green Total Factor Productivity Based on the Spatial Difference-in-Differences Approach: The Case of China

**DOI:** 10.3390/ijerph18179040

**Published:** 2021-08-27

**Authors:** Susheng Wang, Gang Chen, Xue Han

**Affiliations:** 1 School of Economics and Management, Harbin Institute of Technology Shenzhen, Shenzhen 518055, China; wangss@sustech.edu.cn (S.W.); hanxue0960@163.com (X.H.); 2 Department of Finance, Southern University of Science and Technology, Shenzhen 518055, China

**Keywords:** emissions trading system, green total factor productivity, spatial difference-in-difference, energy efficiency, green innovation, industry structure, spatial heterogeneity

## Abstract

How to effectively identify the spatial effect of the emissions trading system(ETS) on urban green total factor productivity(GTFP) generated through the linkage of economic factors between cities is a necessary part of scientifically evaluating the effect of ETS policy in emerging- market countries. This study aims to examine the spatial effect, mechanism, and heterogeneity of the ETS on urban GTFP based on the panel data of 281 cities from 2004 to 2017 in China, applying spatial difference-in-differences(DID) Durbin model (SDID-SDM) with multidimensional fixed effect (FE). The results show that ETS significantly improves the GTFP of the pilot cities, produces a spatial spillover effect and the results are robust to the placebo test, propensity score matching SDID (PSM-SDID) test, and Carbon-ETS interference test. Further analysis shows that the policy effect is mainly driven by improving energy efficiency, promoting green innovation, and optimizing the industrial structure. In addition, we found that ETS performs better in regions with a high degree of marketization, strong environmental law enforcement, and a low proportion of coal consumption. In general, the identification method of this study can be used as a scientific reference for conducting similar research in other emerging countries.

## 1. Introduction

China is the world’s largest developing country and an important member of emerging-market countries. In the past four decades, the gross domestic product (GDP) of China has maintained an average of more than 9%. Even under the negative impact of the 2008 international financial crisis, it has maintained a high growth rate of more than 6.5% (excluding the impact of price factors). However, such rapid economic growth data is accompanied by excessive energy consumption and serious environmental pollution problems [1,2,3]. In 2010, the former Ministry of Environmental Protection, the National Bureau of Statistics, and the former Ministry of Agriculture jointly issued the “First National Pollution Source Census Bulletin” (census in 2010). The communique data shows that the total emissions of major pollutants are 2.32 million tons of sulfur dioxide(SO_2_), 30,289,600 tons of chemical oxygen demand, and 179.777 million tons of nitrogen oxides. To deal with the environmental challenges caused by pollutant emissions, many developed countries such as the European Union and the United States first launched the emissions trading system(ETS), such as the US SO_2_ emission allowance trading project, nitrogen oxides (NO_X_) trading project, etc. [4,5,6]. China formally approved 11 pilot provinces (cities) including Zhejiang, Jiangsu, Inner Mongolia, Hubei, and Hunan as national-level pilot units in 2007 and actively explored and implemented the paid use and transaction system of pollution rights. ETS is an important institutional arrangement aimed at using market mechanisms to reduce pollutant emissions, which can internalize corporate emission reduction costs through total pollutant control and quota trading. The policy goal is to establish a long-term mechanism for energy conservation and emission reduction [7,8]. Therefore, investigating the impact mechanism of the pilot emission trading system on green total factor productivity is crucial to how the government uses market-oriented environmental policy tools to deal with the dual challenges of green sustainable development and high-quality economic growth.

Cities are the main spatial carrier of environmental pollution control, and they are the key to achieving the goal of total pollutant emission, promoting the sound operation of the ETS, and then achieving the goal of green and high-quality development; the environmental regulation goals at the provincial level that are broken down to the enterprise execution level are also implemented at the city level. Due to the circulation connection of resources and factors between cities, the impact of the emission trading system within the city on the emission behavior of enterprises will inevitably produce a certain degree of spatial externality on the surrounding areas through the economic connection between cities [9,10,11]. Nevertheless, previous studies have seldom paid attention to the mechanism of the city-level ETS on the green total factor productivity(GTFP) of the city itself and surrounding areas, and the spatial effect of this market-oriented environmental policy cannot be ignored [12,13].

Theoretically, within the city, the influence of ETS on the territorial GTFP is mainly realized by influencing the decisionmaking of micro-enterprises. Specifically, the pilot policy transfers the cost of emission reduction directly to pollutant producers, who can make flexible choices among pollutant emission quota trading, emission reduction decisions (enhancing energy efficiency, launching green innovation activities, etc.), and location decisions to effectively respond to the policy pressure of the pilot policy [14,15]. In general, when the cost of pollutant emissions is lower than its revenue, these companies choose to purchase pollutant emission quotas; when the revenue from pollutant emissions cannot cover the cost, companies often make active emission reduction decisions such as optimizing resource allocation, improving energy efficiency, and investing in green technologies innovation, etc., for example, when the benefits of green innovation are higher than the cost of emission reduction, companies often choose green technology innovation to solve the pollution problem; and when the benefits of green innovation cannot cover local emission reduction costs, companies often choose to relocate which makes the green innovation benefits of the new location higher than the emission reduction costs. Producers’ emission reduction decisions, green innovation decisions, and relocation decisions at the macro level drive capital and economic factors to gradually withdraw from polluting industries to clean industries, affecting energy efficiency, technological innovation and the adjustment of industrial structure, and then improving the overall GTFP of the city. Among cities, due to the policy pressure of ETS, some emission companies cannot cover emission reduction costs through green innovation and other emission reduction decisions. These companies will choose to withdraw from the local market and relocate. The relocation of these polluting companies can increase the overall GTFP level of the original location. On the other hand, because these companies have technical efficiency advantages relative to the new location, they passively improve the technical efficiency of the new location, thereby generating the spatial spillover effect of GTFP [16].

We use China’s ETS to empirically test the impact mechanism of market-oriented environmental tools on GTFP, which has the following significance: firstly, China’s rapid economic growth is accompanied by serious environmental pollution problems, such as excessive energy consumption and excessive pollutant emissions. These are typical problems that have occurred or are about to occur in developing countries and some emerging-market countries [17]. Secondly, the pilot areas approved by the Chinese government have different geographical locations in which the spatial heterogeneity of humanities, economics, and geography is significant. It is possible to comprehensively examine the possible potential spatial heterogeneity of the effects in areas with different economic development levels and different cultural and geographical characteristics [15]. In addition, the ETS is a typical market-oriented environmental policy tool, and the effect of the policy is sensitive to the degree of marketization in the pilot area and the intensity of environmental law enforcement, and it is a challenge to China, which has been criticized by the West for “low marketization and strong government intervention”. Therefore, it has great practical value to examine the effect of the ETS in the context of government intervention to understand how environmental governance policies improve GTFP in a complex market environment.

In summary, this study is based on the panel data of 281 cities across the country from 2004 to 2017 and takes the spatial difference-in-differences(DID) Durbin model (SDID-SDM) as the benchmark model to empirically examine the impact mechanism of the ETS on GTFP. This study carried out a parallel trend test and found that there is no significant difference between the treatment group and the control group before the implementation of ETS, which means the treatment group and the control group meet the parallel trend assumption. The estimation results of the SDID-SDM model show that the GTFP in the pilot cities has increased significantly, and the ETS also has a positive spatial spillover effect on the GTFP in the surrounding areas of the pilot cities. In addition, to eliminate the potential influence of selectivity bias and possible confounding factors, a series of tests such as placebo test, propensity score matching SDID (PSM-SDID) test, and triple-difference test to exclude the effects of Carbon-ETS were further conducted, and the empirical results remained robust.

In addition, we also conducted empirical tests on the three potential mechanisms of the ETS on GTFP. The mechanism analysis results show that the ETS may achieve the policy effect of improving GTFP by improving energy efficiency, promoting green technology innovation, and optimizing industrial structure. Secondly, by grouping the samples according to the degree of marketization, environmental enforcement, and energy consumption endowment level, we thoroughly investigate the heterogeneity of policy effects. The estimation results show that the positive policy effect of the ETS on GTFP performs better in regions with a high degree of marketization, strong environmental law enforcement, and a low proportion of coal consumption.

The marginal contribution of this research to the existing literature is mainly in the following three aspects: first of all, this study enriches the empirical test of the spatial effect of ETS in improving GTFP. Previous studies were mainly based on the perspective of panel data [18,19,20], ignoring the circulation of resources and factors between cities, and the impact of ETS on corporate emissions behavior will inevitably affect the policy effect through the cross-regional economic connections, which forms the spatial externality of policy effects. It means that the application of panel data model estimation results may lead to model setting bias. This study applies the SDID-SDM approach to estimate the policy effect to avoid this model setting bias which may truly reflect the GTFP promotion effect of the ETS.

Secondly, this study enriches the empirical test of the effectiveness of the ETS in emerging-market countries and developing countries. Previous studies tend to focus on the evaluation of environmental policy effects in developed countries such as in Europe and the United States [15,21], and they easily ignored the effects of environmental policies in countries with poor economic and social development. On the one hand, underdeveloped areas themselves lack the motivation for active market-oriented environmental regulation. On the other hand, it is also related to the reality that underdeveloped regions will undertake the transfer of polluting industries due to the global industrial layout. Developed countries in Europe and the United States have a relatively developed market and economic system and are equipped to effectively implement market-oriented environmental policies. It is worthy of thorough analysis and research on whether emerging-market countries that are under market-oriented construction and developing countries with relatively underdeveloped economic and social development can effectively take market-oriented policy tools to achieve green and sustainable development. Our research results show that even in emerging-market countries and developing countries, market-oriented environmental policy, such as the ETS, can still exert policy effects and bring positive spatial economic effects on GTFP.

Finally, based on the benchmark model, this research further examines the impact mechanism of the ETS on urban GTFP through mechanism analysis and enriches the spatial dimension of the existing ETS mechanism analysis.

The study is organized as follows: Section 2 summarizes the relevant literature and policy implementation background of China’s ETS. Section 3 introduces the empirical research design based on the SDID-SDM. Section 4 reports the estimation results of the benchmark model. Section 5 analyzes the influence mechanism of the policy effect, and Section 6 examines the heterogeneity of the policy effect. Section 7 details the different robustness tests carried out, and the last section gave the main conclusions and policy recommendations of this study on the impact of China’s ETS on GTFP.

## 2. Literature

### 2.1. Green Innovation Effect of ETS

In 1990, Article 4 of the US “Clean Air Act” amendment proposed the “Acid Rain Plan”, which was approved by Congress to use SO_2_ emission allowance trading as a means of emission reduction. It was an early and successful environmental policy tool to achieve reductions through market-based emissions allowance trading [22,23]. Pollutant emission trading refers to the use of emission quota trading to reduce the emissions of major pollutants and reduce the negative impact on the environment. It aims to use market competition and price mechanisms to guide producers to emission reduction behaviors and to achieve the goal of total pollutant control. At the same time, it reduces the overall cost of pollution control in society and realizes green technological innovation. In terms of existing research on the policy effects of the emission trading system, scholars have focused on the following aspects: one is emission reduction effect, which is the core policy goal [24,25]; the other is the economic growth effect, which is the policy economic development goal [26,27]; the third is the green innovation effect which is the efficiency goal, and few scholars have conducted analysis and research on the relationship between the ETS and GTFP [28,29,30]. The first two aspects are the key research content of environmental economics in the past decades, and the green innovation effect of environmental regulations has been the research focus in recent years; of note, the research on the impact of GTFP is becoming an academic hotspot. This study summarizes previous studies on the impact of the ETS on green technology innovation and GTFP, and it shows that the academic community has not reached a consensus on the green innovation effect of the ETS, and the existing research conclusions mainly have three aspects, namely: promotion theory, Inhibition theory and (U-shaped and inverted U-shaped relationship, etc.) nonlinear relationship theory.

First of all, many scholars support the promotion theory based on the classic “Porter Hypothesis” [31,32], that is, environmental policies represented by the ETS have a positive effect on green technology innovation and improves GTFP [33,34]. Zhang L et al. (2019) based on the empirical analysis of the regulatory data of listed companies in seven pilot provinces and cities showed that China(CN)-ETS is significantly positively correlated with green innovation, while market competition has weakened this positive correlation [30]. However, some other scholars in the empirical research found that the classic “Porter hypothesis” conclusion did not appear, and environmental policies brought more negative externalities, that is, environmental policies represented by the ETS restrained the green technological innovation and hindered the green technological innovation process of enterprises [35,36]. In addition, some studies found that both the promotion theory and the suppression theory are valid. They are just the results of different effects shown in different stages of nonlinear policy effects. Therefore, the impact of environmental policies represented by the ETS on GTFP is a process of non-linear change with both promotion and suppression effects [20].

In summary, the research on the impact of ETS on GTFP is important, but the academic community has not yet reached a consensus. Moreover, the sample areas for this type of research are mostly developed countries such as regions in Europe and the United States, and they lack extensive attention to relatively underdeveloped economies such as emerging-market countries and developing countries. However, it is precisely these relatively underdeveloped economies that are facing more complex environmental issues. In recent years, scholars have begun to pay attention to the treatment effects of market-oriented environmental policy tools in emerging-market countries and developing countries [37,38,39], such as Oliveira et al. (2019), who applied the Economic Projection and Policy Analysis (EPPA6) model to assess ETS cooperation between Brazil and Europe. This showed that a domestic ETS reduces emissions and promotes technological substitution towards alternative energy for both participants [40]. Therefore, we use China’s ETS as a research sample which is a typical representative of emerging-market countries and developing countries, to examine the identification of the policy effects of the ETS. It will help to investigate whether economies facing the dual pressure of economic growth and sustainable development can adopt market-oriented environmental policy tools to a green effect, and how the influence mechanism works on the green economy effect.

### 2.2. ETS in China

**Figure 1:**
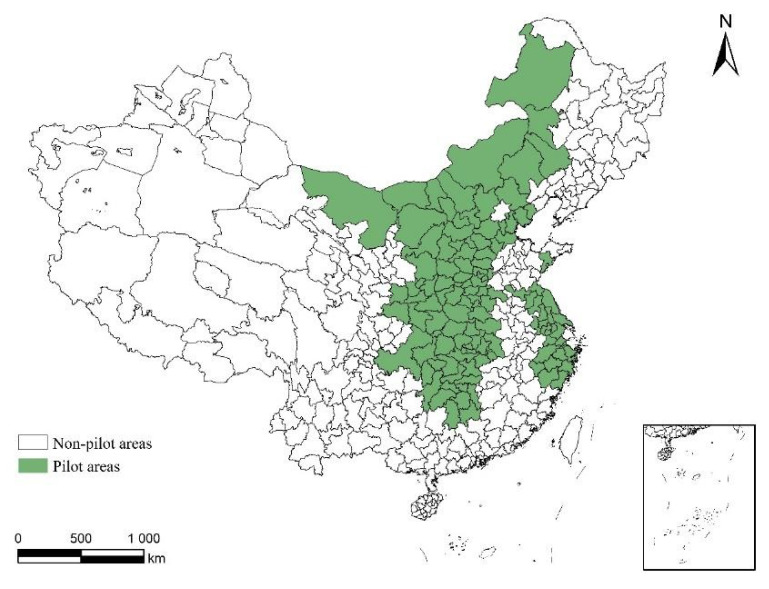
Distribution of ETS pilot areas in China.

To reverse the extensive economic growth mode with high pollution, high energy consumption, high emissions, and low efficiency and to quickly realize the sustainable development of a green economy, the Chinese government has conducted a great deal of environmental policy exploration, especially about the issue of pollutant emissions [41,42]. In 1987, the Minhang District of Shanghai launched a water pollutant discharge transaction. In 1988, the former National Environmental Protection Agency approved 18 cities including Shanghai, Beijing, Tianjin, Shenyang, Xuzhou, and Changzhou as pilot areas developing water pollution discharge permits. In 2003, the former State Environmental Protection Administration cooperated with the US Environmental Protection Association to carry out training on total sulfur dioxide control and emissions trading across the country. In 2007, the Ministry of Finance together with the former Ministry of Environmental Protection and the National Development and Reform Commission successively approved 11 provinces (cities) including Tianjin, Hebei, Shanxi, Inner Mongolia, Jiangsu, Zhejiang, Henan, Hubei, Hunan, Chongqing and Shaanxi as national pilot units to carry out the pilot work of the ETS. In 2014, Qingdao City of Shandong Province was included in the pilot program (see Figure 1). The emission trading training in cooperation with the U.S. Environmental Protection Association makes China’s ETS design similar to that of the United States, including total amount control, initial allocation, quota period, quota use, and penalties for violations, while the specific content is subject to adaptive adjustments by the local government which are significant regional differences, such as transaction methods (competitive transactions, negotiated transactions, public auctions, quota transfers, etc.) and transaction price systems (paid use prices, transaction benchmark prices, government repurchase prices).

Based on the statistical data officially released by China, the ETS in China has achieved its policy results. The first is the effect of pollutant emission reduction. The “Second National Pollution Source Census Bulletin” jointly issued by the three departments in 2020 (the census period is 2017) shows that compared with the data of the first national pollution source census, the emissions of pollutants in 2017 such as the sulfur dioxide, chemical oxygen demand, and nitrogen oxides decreased by 72%, 46%, and 34% respectively compared with 2007. The second is the scale of emissions trading. As of August 2018, the total amount of paid use fees for emission rights collected on the primary market was 11.77 billion yuan, and the cumulative transaction amount in the secondary market was 7.23 billion yuan. It shows that China’s pilot policy for emissions trading has achieved goals in pollutant reduction and economic benefits, but official statistics have not disclosed the green innovation effect of the pilot policy, so it is necessary to conduct a more in-depth analysis to examine whether the pilot policy achieves the policy goal of improving GTFP and to deeply explore the internal mechanism of the pilot policy affecting GTFP and the heterogeneity of policy effects.

## 3. Research Design

### 3.1. Samples and Data

In 2007, the Ministry of Finance, the former Ministry of Environmental Protection, and the National Development and Reform Commission successively approved 11 provinces (cities) including Tianjin, Hebei, Shanxi, Inner Mongolia, Jiangsu, Zhejiang, Henan, Hubei, Hunan, Chongqing and Shaanxi as national pilot units to carry out the pilot work of the ETS. In 2014, Qingdao City in Shandong Province was included in the pilot program. This study uses 2003–2017 as sample period, deleting the cities adjusted by administrative divisions during the sample period, and replacing them with newer administrative divisions, such as Chaohu City in Anhui Province and Longnan City in Gansu Province, also deleting areas with missing data such as Tibet et al. In addition, Hong Kong, Macao, and Taiwan, all with different statistical calibers, were deleted. Finally, the panel data of 281 cities from 2003 to 2017 were selected as the research data set for empirical testing. Economic data at the city level were deflated based on prices in 2003. The input-output data required for GTFP calculations including undesired output and control variable data are all from the “China Statistical Yearbook”, “China Regional Economic Statistical Yearbook”, and “China City Statistical Yearbook”. The city patent authorization data comes from the China Research Data Service Platform (CNRDS) database.

### 3.2. Variables

#### 3.2.1. Dependent Variable—GTFP

The measurement of GTFP is mainly based on the malmquist-luenberger(ML) index method of the slacks-based measure(SBM) directional distance function [43]. The input factors include labor (employed population), capital (capital stock), and energy (industrial electricity). The expected output is the actual GDP of the region after price deflation. The undesired output is industrial smoke and dust emissions (tons), wastewater emissions, sulfur dioxide emissions (tons), and PM 2.5.

#### 3.2.2. Key Explanatory Variable—ETS Dummy

The key explanatory variable of this study is the multi-period combined dummy variable of the ETS, which is composed of the pilot group dummy variable and the pilot time group dummy variable. When a city belongs to the pilot group, the pilot group dummy variable is 1; otherwise, it is 0. The pilot cities include 12 provinces (cities), which are Tianjin, Hebei, Shanxi, Inner Mongolia, Jiangsu, Zhejiang, Henan, Hubei, Hunan, Chongqing, Shaanxi, and Shandong Qingdao, that is, there are 109 cities in the treatment group, and the remaining 172 cities are the control group. In addition, since the approval time of the above 109 pilot cities is not uniform and belongs to the multi-phase DID situation, it is assumed that when city *i* is approved as a pilot in year *t*, the value of the city will be 1 for each subsequent year (because the months of approval for the pilot project are all at the end of the current year, so the second year of approval is used as the starting point of the pilot. For example, Qingdao City in Shandong Province was approved as a pilot in December 2014, so 2015 is the starting year of the Qingdao pilot.).

#### 3.2.3. Control Variables

Based on existing literature [18,20], we controlled for a set of variables to capture the influence factors of the GTFP. The control variables mainly include economic development level (measured by per capita GDP), population size (measured by population density), energy consumption scale (measured by industrial electricity consumption), industrial structure (measured by industrial output value to GDP), and innovation level (measured by the number of invention patents).

### 3.3. Empirical Model

We applied SDID-SDM to study the impact of ETS on GTFP. The SDID-SDM model is setting as follows:

(1) $$gtfp_{it}=\alpha_{0}+\rho {\left(\Omega {}^{\prime }⊗W{}^{\prime }\right)}_{it}gtfp_{it}+\alpha_{1}DID_{it}+\gamma_{1}{\left(\Omega {}^{\prime }⊗W{}^{\prime }\right)}_{it}DID_{it}+\gamma_{2}{\left(\Omega {}^{\prime }⊗W{}^{\prime }\right)}_{it}control_{it}+\alpha_{2}control_{it}+city_{i}+year_{t}+\varepsilon_{it}$$

where the dependent variable *gtfp_it_* denotes the GTFP measured by SBM-ML in city *i* at year *t*. the independent variable *DID_it_* denotes the dummy variable of ETS, which equals 1 if the city *i* at year *t* is approved as the pilots; otherwise, it equals 0. *control_it_* denotes control variables. ${\left(\Omega {}^{\prime }⊗W{}^{\prime }\right)}_{it}$ denotes the space-time weight matrix, while *Ω*′ denotes the temporal weight matrix, and *W*′ denotes the spatial weight matrix. *α*_0_ denotes the constant, *ρ* denotes the coefficients of spatial lag of dependent variable, *α*_1_ denotes the coefficients of the independent variable, *α*_2_ denotes the coefficients of control variables, *γ_1_* denotes the coefficients of spatial lag of independent variable, and *γ*_2_ denotes the coefficients of spatial lag of control variable. *city_i_* is urban fixed effects absorbing all unobserved city-specific, time-invariant factors that may influence the dependent variable. *year_t_* is the year fixed effect to control for the general macroeconomic factors affecting all cities. *ε_it_* is a random error.

## 4. Empirical Analysis

### 4.1. Parallel Trend Test

**Figure 2:**
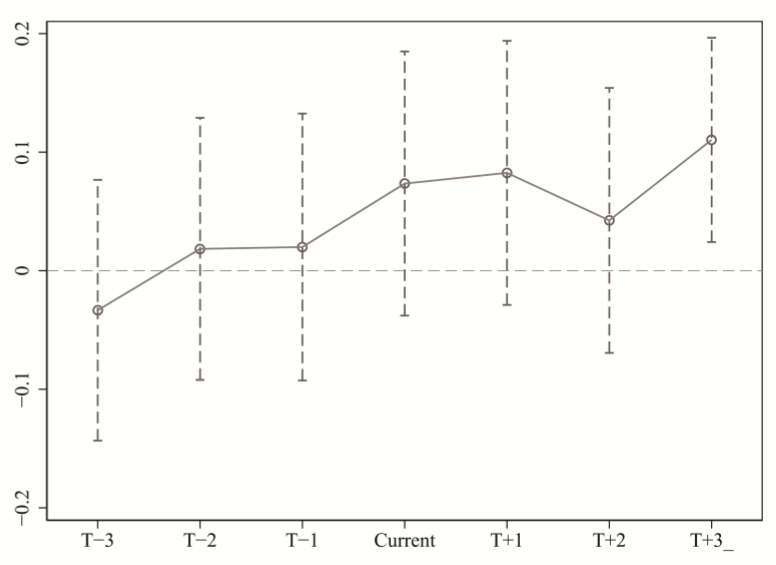
Parallel trend test.

The key identification hypothesis of the DID model is that non-pilot areas provide effective counterfactual changes for the policy treatment effects of pilot areas [44,45], that is, before the implementation of the ETS, urban GTFP maintained relatively stable changes, while there is a significant difference between the treatment group and the control group after the pilot implementation. To ensure the basic assumption is met, this study follows the parallel trend test method of multi-period DID, and performed SDID-SDM regression for the first three years and the last three years of the treatment period. The regression results show that before the implementation of the emission trading system, there was no systematic difference in the time trend between the pilot area and non-pilot area which means it satisfies the parallel trend assumption. It should be noted that there is a certain time lag effect in the emission trading system, as shown in Figure 2, three years after the treatment time.

### 4.2. Baseline Regression

As shown in Table 1, column (1) is the estimated result of the panel-DID model as a comparison to investigate the average treatment effect when there is no spatial dimension, and column (2) is the estimated result of Equation (1). The results show that after controlling for city-fixed effects, year-fixed effects, and control variables, without considering spatial effects, the ETS has a significant positive effect on GTFP in pilot cities. After considering the spatial effects in this study, the ETS still significantly improves the GTFP of the pilot cities, and the coefficient is significant at the 1% confidence level. In addition, the ETS also drives the growth of GTFP in the surrounding areas of the pilot cities, and the spatial lag coefficient is significant at the 5% confidence level. This finding is consistent with existing relevant research conclusions and theoretical hypotheses [18,20,46], that is, after the implementation of the ETS, the GTFP of the territorial cities and surrounding areas increased significantly.

## 5. Mechanism Analysis

As discussed in Section 1, the ETS may affect GTFP changes through energy efficiency, green innovation, and industrial structure. We empirically tested these potential impact mechanisms respectively.

### 5.1. Impact of Energy Efficiency

When faced with the policy pressure of the emission trading system, producers in pilot cities often choose different emission strategies based on the relationship between emission quota expenditures and emission benefits and between emission reduction costs and emission reduction benefits. When the emission quota expenditure is higher than the emission income, producers will adopt corresponding energy-saving and emission-reduction measures under policy pressure, such as improving energy efficiency by reforming energy technology and changing energy consumption structure [47]. The improvement of energy efficiency can significantly improve the allocation efficiency of energy resources, thereby realizing the improvement of GTFP. In addition, there are still some companies that cannot cover costs even if they improve energy efficiency. These producers often choose green technology innovation or relocation. Relocation will not only improve the overall energy efficiency level of the original location but also relies on the energy efficiency advantages of the original location relative to the new location to indirectly improve the energy efficiency level of the new location, and then turns out a positive spatial spillover effect. We used the ratio of industrial electricity consumption to regional GDP, that is, the level of energy consumption per unit of GDP, to measure city-level energy efficiency and examine the mechanism through the following model:

(2) $$\begin{array}{l} gtfp_{it}=\alpha_{0}+\rho {\left(\Omega {}^{\prime }⊗W{}^{\prime }\right)}_{it}gtfp_{it}+\gamma_{2}{\left(\Omega {}^{\prime }⊗W{}^{\prime }\right)}_{it}control_{it}+\alpha_{2}control_{it}+city_{i}+year_{t}+\varepsilon_{it}\\ +(\alpha_{1}DID_{it}+\alpha_{3}ee_{it}+\alpha_{4}DID_{it}\times ee_{it})+{\left(\Omega {}^{\prime }⊗W{}^{\prime }\right)}_{it}(\gamma_{1}DID_{it}+\gamma_{3}ee_{it}+\gamma_{4}DID_{it}\times ee_{it}) \end{array}$$

Table 2 column (1) gives the estimated results of Equation (2). It is shown that the interaction coefficient of the emission trading pilot DID and energy efficiency is significantly positive at the 5% level, and the spatial lag coefficient of the interaction term is significantly positive at the 10% level, indicating that the emission trading system can improve the energy efficiency of territorial cities, and it can also positively promote GTFP in the surrounding areas of the pilot cities.

### 5.2. Impact of Green Innovation

The impact of the ETS on green technology innovation is mainly reflected in two aspects. On the one hand, the classic Porter hypothesis, strict and flexible environmental regulations can provide incentives for clean technology innovation ([48]). The ETS will not only stimulate quota income companies to obtain higher quota sales profits through innovation but also stimulate emission companies that purchase quotas to reduce pollution costs and increase profits through green innovation. These two ways work together to enhance the overall green technological innovation of the city. On the other hand, for those companies that are unwilling to innovate or have low innovation gains, they will relocate the companies from the pressure area through relocation decisions, thereby improving the green efficiency in the region, and the companies that move away will also increase the technological efficiency of the new location. Therefore, it produces the spatial spillover effect of green innovation, which we call the Porter spatial effect. We use the ratio of the number of green patents to the total number of patent grants to measure the level of green innovation at the city level and examine the above mechanism through the following model:

(3) $$\begin{array}{l} gtfp_{it}=\alpha_{0}+\rho {\left(\Omega {}^{\prime }⊗W{}^{\prime }\right)}_{it}gtfp_{it}+\gamma_{2}{\left(\Omega {}^{\prime }⊗W{}^{\prime }\right)}_{it}control_{it}+\alpha_{2}control_{it}+city_{i}+year_{t}+\varepsilon_{it}\\ +(\alpha_{1}DID_{it}+\alpha_{3}gti_{it}+\alpha_{4}DID_{it}\times gti_{it})+{\left(\Omega {}^{\prime }⊗W{}^{\prime }\right)}_{it}(\gamma_{1}DID_{it}+\gamma_{3}gti_{it}+\gamma_{4}DID_{it}\times gti_{it}) \end{array}$$

Table 2 column (2) gives the estimated results of Equation (3). The interaction coefficient of the emission trading pilot DID and green innovation is significantly positive at the 10% level, and the spatial lag coefficient of the interaction term is significantly positive at the 10% level, indicating that the emission trading system can improve the green innovation of territorial cities, and it can also positively promote GTFP in the surrounding areas of the pilot cities which verifies the Porter spatial effect of the ETS.

### 5.3. Impact of Industry Structure

The ETS can also affect the macro-industrial structure of cities [49] and affect GTFP through industrial structure adjustments. In the pilot areas, due to severe environmental protection policy pressures, operating costs of pollutant producers have significantly increased, such as the cost of pollutant emission quotas, pollutant reduction cost, monitoring and verification cost of pollutant emissions, etc., which directly or indirectly affect the resource allocation decision of the enterprise which may gradually fade out of polluting industries; this will reduce the proportion of the secondary industry [15,50]. In addition, the total emission limit and the market-oriented quota trading mechanism will promote the gradual transfer of pollution capital from the pollution industry to the clean industry or other industries, promote the green innovation of the pollution industry, and further accelerate the shrinkage of the traditional pollution industry. Therefore, the ETS can theoretically affect GTFP by affecting the adjustment of the industrial structure. We use the proportion of the secondary industry to measure the city-level industrial structure, and the estimation model is set as follows:

(4) $$\begin{array}{l} gtfp_{it}=\alpha_{0}+\rho {\left(\Omega {}^{\prime }⊗W{}^{\prime }\right)}_{it}gtfp_{it}+\gamma_{2}{\left(\Omega {}^{\prime }⊗W{}^{\prime }\right)}_{it}control_{it}+\alpha_{2}control_{it}+city_{i}+year_{t}+\varepsilon_{it}\\ +(\alpha_{1}DID_{it}+\alpha_{3}str_{it}+\alpha_{4}DID_{it}\times str_{it})+{\left(\Omega {}^{\prime }⊗W{}^{\prime }\right)}_{it}(\gamma_{1}DID_{it}+\gamma_{3}str_{it}+\gamma_{4}DID_{it}\times str_{it}) \end{array}$$

Table 2 column (3) gives the estimated results of Equation (4). The interaction coefficient between the DID and the industrial structure is significantly negative at the 5% level, and the spatial lag coefficient of the interaction term is significantly positive at the 10% level, indicating that ETS can increase urban GTFP by reducing the proportion of the secondary industry in pilot cities, and it can also increase the GTFP in the surrounding areas by increasing the proportion of the secondary industry in the surrounding cities to produce a positive spatial spillover effect.

## 6. Heterogeneity Analysis

The effective implementation of market-based environmental governance policies depends on the pilot areas with a high level of marketization. When the pilot areas have a low level of marketization, a market-based environmental policy, such as ETS, will be greatly restricted and then unable to handle the internalization of pollution emissions cost, and the quota revenue cannot cover the cost of reduction well, which makes the policy effect greatly discounted [18]. Therefore, the treatment effect of the ETS will show spatial heterogeneity due to the different degrees of regional marketization. Secondly, the effective implementation of market-oriented environmental policies is also closely related to the implementation of regional environmental enforcement [51,52]. Strong environmental enforcement by local governments will improve environmental governance efficiency. As known, the environmental enforcement of environmental policies in developing countries and some emerging-market countries is weak, and previous studies have shown that the effectiveness of environmental policy is greatly reduced in areas where environmental enforcement is low (poor supervision). Regulatory power rent seeking is an important cause of environmental economic corruption that leads to weak environmental enforcement [53]. In addition, the region’s energy consumption endowment (mainly the proportion of coal consumption) is also an important source of the spatial heterogeneity of environmental policy effects [54].

According to the research of Li R, Ramanathan R (2018, [55]), Hou B et al. (2020, [18]), we adopt the provincial marketization data disclosed by Fan Gang et al. (2011, [56]), according to the median of the total marketization index divides the sample cities into two groups, namely, regions with a higher degree of marketization and regions with a lower degree of marketization. Secondly, different from Hou B et al. (2020, [18]), this study used the ratio of the number of environmental administrative punishment cases to the total energy consumption in the provincial environmental law enforcement data disclosed in the “China Environmental Statistics Yearbook” to measure the intensity of regional environmental enforcement. This measurement can avoid differences in environmental enforcement caused by differences in energy consumption between provinces. Cities are also grouped according to the median of the number of cases and divided into two groups: strong law enforcement and weak law enforcement. Similarly, we used provincial energy structure data to measure urban energy consumption endowment, which is measured by the proportion of provincial coal consumption in energy consumption based on the data from the “China Energy Statistics Yearbook”, to group it by the median, and classify it into high coal consumption group and low coal consumption group. It should be noted that the above three data sources are all province-level data. It is reasonable to use province-level data to assign value to cities for which spatial heterogeneity of China’s environmental enforcement and marketization is mainly driven by provincial differences because differences in culture, economic development, and energy consumption endowments within the province are generally significantly smaller than inter-provincial differences. The groupings in the heterogeneity analysis all adopted the data before the pilot period which may somehow avoid possible selectivity deviations, and in this study, we adopted the data from 2007.

Table 3 shows the estimated results of the heterogeneity analysis of policy effects. Columns (1)–(2) are the estimated results of different marketization levels. Coefficients in column (1) are significant, and column (2) is not significant, indicating that the average treatment effect of the pilot policies in regions with a high degree of marketization on GTFP is still significant, and the ETS in regions with a low degree of marketization have no significant impact on GTFP. Secondly, the coefficients of the DID and W*DID in column (1) are higher than those of column (2), indicating that the pilot program in regions with a higher degree of marketization performs better. This heterogeneous result is relatively easy to understand. The ETS is a typical market-oriented environmental policy, and the degree of marketization of the pilot city will directly affect the effectiveness of the pilot policy on market. The higher the marketization, the clearer the pilot policy signals will be communicated, and the easier it will be to influence enterprises’ pollution discharge decisions, green innovation decisions, and relocation decisions through policy pressures, resulting in better effects on GTFP.

Columns (3)–(4) are the estimated results of different environmental enforcement. The coefficient of the DID in column (3) is not significant, the coefficient of the W × DID is significant, and the estimation results in column (4) are significant. It shows that the ETS in different environmental enforcement groups still significantly affects GTFP, and there are differences between groups in treatment effects. It shows that the average treatment effect of the regional pilot policies with strong environmental enforcement on GTFP is mainly achieved through negative spatial externalities, while the impact of the regional pilot policies with weak enforcement on GTFP is consistent with the benchmark model. Secondly, the coefficient of the W × DID in column (3) is negative, which may indicate that areas with strong environmental law enforcement will increase the burden of environmental PR costs for companies in the pilot city. Compared with areas with weak law enforcement, more companies will consider relocation decisions, and these relocation companies only relocate due to the strong environmental enforcement rather than the pressure from ETS, which makes the new location have a green productivity advantage over the old location. Therefore, this relocation decision will increase the GTFP of the old location while reducing that of the new location. The objective reason is that excessively strict environmental enforcement has distorted the market-oriented allocation process of the ETS.

Columns (5)–(6) are the estimation results of different energy consumption endowments. The coefficients of columns (5)–(6) are all significant. The coefficient of DID in column (5) is significantly lower than column (6), the W*DID coefficient in column (5) is positive, and the W*DID coefficient in column (6) is negative, which means there are significant differences between the group. The DID coefficient in column (5) is significantly lower than that in column (6). It indicates that the effect of the ETS in areas with a higher proportion of coal consumption is significantly lower than that in areas with a lower proportion of coal consumption, which means that pilot cities in regions with the lower coal consumption are more inclined to use clean technologies (non-fossil energy) for production, so producers’ emission reduction decisions are more inclined to improve energy efficiency, carry out green innovation activities, etc., which then more significantly improve productivity. Moreover, these tendencies to clean technology indicate that pilot cities in regions with a low proportion of coal consumption have higher innovation gains. It will attract companies with higher green productivity in surrounding cities to move to pilot cities, which will cause negative spatial externality, so the coefficient of the W*DID in column (6) is negative. The effects of pilot policies in areas with a higher proportion of coal consumption are different. Producers in high coal consumption areas tend to make emission reduction decisions in improving energy efficiency while purchasing emissions quota. The green innovation income of these producers is relatively weaker than the first two, and the effect is consistent with the benchmark model, that is, it positively promotes GTFP in territorial cities and has a positive spatial spillover effect on surrounding cities.

Above all, we believe that the treatment effect of the ETS on GTFP shows significant heterogeneous characteristics under different marketization levels, environmental enforcement efforts, and energy consumption endowments, and the promoting effects perform better in regions with high marketization, strong environmental enforcement, and low coal consumption.

## 7. Robustness Test

### 7.1. Placebo Test

**Figure 3:**
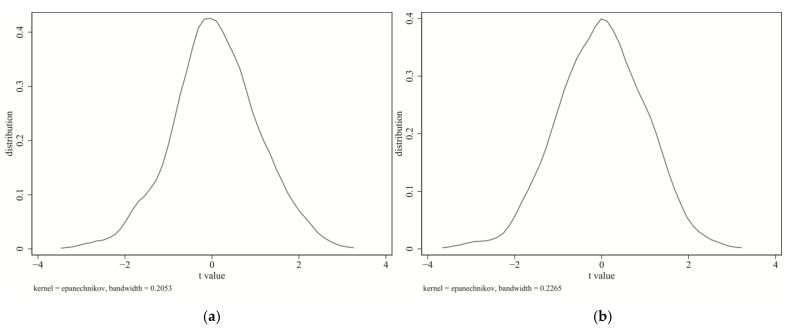
Placebo test. (**a**) shows the coefficient’s *t* distribution of *DID* calculated in the placebo test; (**b**) shows the coefficient *t* distribution of *W* × *DID* calculated in the placebo test.

To further eliminate the influence of other unknown factors on the selection of pilot cities, this study conducted 999 samplings in all 281 cities and randomly selected 108 cities as the virtual treatment group for each sampling (the original number of treatment groups was 108), and the remaining 173 cities were used as a randomized control group. We estimated the placebo test by adopting the SDID-SDM approach, and the results are as shown in Figure 3. Figure 3a,b are, respectively, the distribution diagrams of the coefficients of DID and WDID in the random sampling estimation results. It can be found that the *t* value of most sampling estimation coefficients changes within a small range, and the significance fails (that is, the pass rate is still low even under the 10% confidence level), indicating that the ETS did not show a significant treatment effect in the random sampling simulation. It can be considered that the conclusion of the treatment effect identified by the benchmark model estimation passed the placebo test.

### 7.2. PSM-SDID

Although the ETS has a significant promotion and positive spatial spillover effect on GTFP, the result may be caused by potential selectivity bias [49]. Therefore, we used the propensity score matching (PSM) method to solve the problem of selective bias that may exist in the grouping by identifying and matching to form a new treatment group and control group and then continued to use the SDID-SDM model to identify and evaluate the treatment effect. The results of the estimation of the PSM-SDID method are shown in Table 4 (1). This study found that the ETS still significantly improves the GTFP of the territorial cities and has a positive spatial spillover effect on the GTFP of the surrounding cities, indicating that the research conclusions of the benchmark model are robust.

### 7.3. Carbon ETS

Many existing policy studies on the carbon ETS have used the ETS as a confusing factor. These studies control the possible confusion caused by the ETS by constructing dummy variables of the ETS [18]. Therefore, in contrast to these studies, this study constructed a combined dummy variable for the carbon ETS, that is, during the treatment period, the value of the dummy variable of carbon emission trading pilot is 1but otherwise 0. Then we bought it into the benchmark SDID-SDM model, and adopted the difference-in difference-in-differences(DDD) method to identify and estimate the model. The results are shown in column (2) of Table 4. After controlling the confusing effects of the carbon ETS, the ETS still significantly increases the GTFP of the territorial cities and has a positive spatial spillover effect on the GTFP of the surrounding cities, indicating that the results of the policy effects are robust.

## 8. Discussion and Conclusions

### 8.1. Discussion

To accelerate the transition from extensive economic growth to green and high-quality development, the Chinese government has carried out more environmental policy explorations. Among them, the market-oriented environmental policy tool ETS has achieved remarkable green economic benefits. Adopting the SDID-SDM model with multidimensional FE, we examined the spatial policy impact of ETS on urban GTFP.

Benchmark regression results showed that, after controlling for individual fixed effects, time fixed effects, and the influence of control variables, the ETS policy implemented by China has effectively increased the GTFP of territorial cities, which is consistent with the existing research conclusions [18,20]. In addition, as mentioned above, the effect of ETS policy will accompany the commodity trade between cities and the circulation of resource elements to produce spatial correlation, which is ignored or less mentioned in previous studies [20,30]. Ignoring the impact of inter-city spatial interactions on policy effects will bias the identification results of ETS policy effects. In this regard, we effectively identified the spatial effects of ETS policies by applying the SDID-SDM model.

Subsequently, we also discussed in detail how ETS influences urban GTFP through energy efficiency, green technology innovation, and industrial structure and the identification of spatial effects under the corresponding mechanism. This verifies the important role of urban spatial connections in energy efficiency improvement, green innovation, and industrial structure adjustment [57]. The possible explanation behind this is that we believe it is related to the location decision making of pollutant companies. The research on heterogeneous enterprise location selection theory shows that enterprise location selection will directly or indirectly affect regional efficiency, regional innovation, regional industrial structure, and regional spatial structure [58], and the policy pressure of ETS will drive some pollutant companies to relocate. The spatial redistribution process of a large number of pollutant companies will promote ETS policy effects to show more spatial effects through macro-level energy efficiency, green technology innovation, and industrial structure.

In addition, the heterogeneity analysis results show that the level of marketization, environmental law enforcement, and regional energy consumption endowments are important sources of heterogeneity in the effectiveness of ETS policies. Firstly, a higher level of market-oriented reform will bring about a more obvious effect of improving territorial GTFP and the spatial spillover effect of surrounding GTFP. The policy effect of lower regions is not significant. This result on the one hand confirms that China’s market reform has achieved certain results and refutes the doubts of some foreign scholars [20,59]. It also shows the spatial heterogeneity of China’s market-oriented reforms. In the future, China needs to continue to explore market-oriented reforms in areas with low levels of marketization and further guide the market to play an important role in the allocation of resource elements. At the same time, it also means that China should extend the successful experience of emissions trading pilots to more regions; expand other possible market-oriented environmental policy tools, including intertemporal emissions rights, cross-regional trade, and green financial instrument design, etc.; accelerate the improvement of the regional green policy system; actively integrate into the international environmental governance system; learn from the advanced environmental governance experience of developed countries; and enhance the right to speak in international environmental governance.

Secondly, areas with strong environmental law enforcement have a negative siphon effect, and areas with low environmental enforcement have a positive spillover. This result confirms that the difference in supervision will have opposite spatial effects. When the supervision is strong, the companies that choose to actively respond to the strategy of polluting companies will benefit more, which will form a trend of gathering in regions with strong supervision, and those pollutant companies that respond negatively will spread to the surrounding area and then cause a negative siphon effect. On the contrary, in regions with loose supervision, the profits of pollutant companies that adopt active strategies have dropped significantly. More companies choose to purchase pollutant emission rights for negative response strategies such as pollutant emission. In turn, innovative enterprises move abroad, and there is a positive spatial spillover effect. In this regard, we propose the following suggestions: one is to strengthen the supervision of local government environmental law enforcement departments [60], take a zero tolerance stance for environmental corruption, increase the rent-seeking cost of local government officials’ environmental supervision powers and avoid the harmful impact of environmental economic corruption on the burden of enterprises; the other is to increase the transparency of the environmental law enforcement process, make the law enforcement process open and transparent, and disclose information on law enforcement documents.

Finally, the heterogeneity brought about by the endowment of urban energy consumption shows that coal consumption has a certain path dependence. To break this path, we must start with the energy consumption structure, use compulsory or semi-compulsory policy tools to guide areas with a high proportion of coal consumption to carry out energy efficiency improvement and green innovation activities and guide the use of clean technology.

### 8.2. Conclusions

In general, our research provides empirical support for the significant spatial effects of ETS’s impact on urban GTFP and shows that the spatial correlation between cities has significant spatial effects in terms of policy effect, influence mechanism, heterogeneity, etc., which makes up for the neglect of the existing ETS policy effect identification research on the spatial dimension. In addition, the identification method of this study can be used as a scientific reference for conducting similar research in other emerging countries.

Although this research provided some valuable findings and enlightenment for the government’s decision making and research in the field of emission reduction and green growth, it inevitably has certain limitations. First of all, our research only takes China’s ETS as a case. No cases of other emerging-market countries were introduced, and there is a lack of more extensive identification verification. Secondly, there is a lack of finer-grained analysis on the heterogeneity of policy spatial effects. For example, the introduction of spatial measurement technologies such as multiscale geographically weighted regression(MGWR) and geographical and temporal weighted regression (GTWR) allows finer-grained space correlation effects, which will provide help for the precise identification of policy effects. Finally, we did not conduct a more in-depth analysis of areas with weak environmental law enforcement and did not examine in detail the possible role and influence mechanism of corruption in the heterogeneity of spatial effects.

## Figures and Tables

**Table 1:** Baseline regression results.

Model	Panel-DID	SDID-SDM
Variables	(1)	(2)
*DID*	0.491 ***	0.662 ***
	(11.57)	(2.93)
*W* × *DID*		2.190 **
		(2.11)
*Control*	Y	Y
*Year-fe*	Y	Y
*City-fe*	Y	Y
*Obs.*	3934	3934
*R* ^2^	0.440	0.472

Note: DID is short for difference-in-differences, SDID-SDM is short for spatial difference-in-differences Durbin model. The parentheses are the *t*-values. *** and ** represent significant levels at 1% and 5%, respectively.

**Table 2:** Results of the impact mechanism analysis.

Model	Energy Efficiency	Green Innovation	Industry Structure
Variables	(1)	(2)	(3)
*DID* × *ee*	0.036 **		
	(2.21)		
*W* × *DID* × *ee*	0.413 *		
	(1.65)		
*DID* × *gti*		0.280 *	
		(1.83)	
*W* × *DID* × *gti*		3.725 *	
		(1.91)	
*DID* × *str*			−0.295 **
			(−2.26)
*W* × *DID* × *str*			1.857 *
			(1.75)
*Control*	Y	Y	Y
*Year-fe*	Y	Y	Y
*City-fe*	Y	Y	Y
*Obs.*	3934	3934	3934
*R* ^2^	0.356	0.359	0.355

Note: DID is short for difference-in-differences. The parentheses are the *t*-values. ** and * represent significant levels at 5%, and 10%, respectively.

**Table 3:** Heterogeneity analysis results.

Model	Marketization Level		Environmental Enforcement		Energy Consumption Endowment	
	(1)	(2)	(3)	(4)	(5)	(6)
Variables	High	Low	Strong	Weak	Heavy	Light
*DID*	0.758 ***	0.014	0.680	0.433 ** (2.29)	0.611 ***	11.394 ***
	(3.08)	(0.03)	(1.06)		(2.74)	(5.25)
*W* × *DID*	3.189 ***	2.496	−6.246 ***	6.064 * (1.958)	2.732 **	−6.434 ***
	(3.76)	(1.20)	(−2.77)		(2.24)	(−2.59)
*Control*	Y	Y	Y	Y	Y	Y
*Year-fe*	Y	Y	Y	Y	Y	Y
*City-fe*	Y	Y	Y	Y	Y	Y
*Obs.*	2268	1666	2240	1694	2450	1484
*R* ^2^	0.434	0.501	0.486	0.519	0.412	0.536

Note: DID is short for difference-in-differences. The parentheses are the *t*-values. ***, ** and * represent significant levels at 1%, 5%, and 10%, respectively.

**Table 4:** Estimation results in PSM-SDID and effect with Carbon ETS.

Model	PSM-SDID	Carbon ETS
Variables	(1)	(2)
*DID*	0.640 ***	
	(2.84)	
*W* × *DID*	3.110 **	
	(2.56)	
*DDD*		0.195 *
		(1.87)
*W* × *DDD*		43.441 ***
		(6.63)
*Control*	Y	Y
*Year-fe*	Y	Y
*City-fe*	Y	Y
*Obs.*	3201	3934
*R* ^2^	0.150	0.271

Note: DID is short for difference-in-differences, PSM-SDID is short for propensity score matching spatial difference-in-differences, ETS is short for emissions trading system, DDD is short for difference-in- difference-in-difference. The parentheses are the t-values. ***, ** and * represent significant levels at 1%, 5%, and 10%, respectively.

## Data Availability

Data available on request due to restrictions privacy. The data presented in this study are available on request from the corresponding author. The data are not publicly available due to privacy.
